# Update of the pediatric hypotension graphic adjusted for gender and height percentiles: diastolic blood pressure for boys, 1 to 17 years old

**DOI:** 10.1186/cc12636

**Published:** 2013-06-19

**Authors:** HH Shieh, ER Barreira, A Bousso, AC Ventura, EJ Troster

**Affiliations:** 1 University Hospital of Universidade de São Paulo, Vila Iara, São Paulo, SP, Brazil

## Introduction

According to the National Heart, Lung, and Blood Institute of the National Institute of Health, hypotension refers to an abnormally low blood pressure (BP). In childhood, hypotension can be determined according to two different definitions: BP below the 5th percentile or below two standard deviations (SDs) of the mean for age and gender [1]. A graphic representation of pediatric hypotension was published in 1977 [2], and no updates have been published since then.

The objective of this study was to update the graphic representation of pediatric hypotension.

## Methods

We used a computerized calculation method to develop high-resolution graphics containing 5,841 points each, to depict the main percentiles associated with low BP for boys from 1 to 17 years old in the 50th percentile of height. Each point represents the calculation of the polynomial equation that includes the statistical processing of the last Report on Blood Pressure [3]. We also analyzed the effect of height on BP in the 5th to 95th percentile range. Statistical functions generated by computerized program were used.

## Results

**Figure 1:**
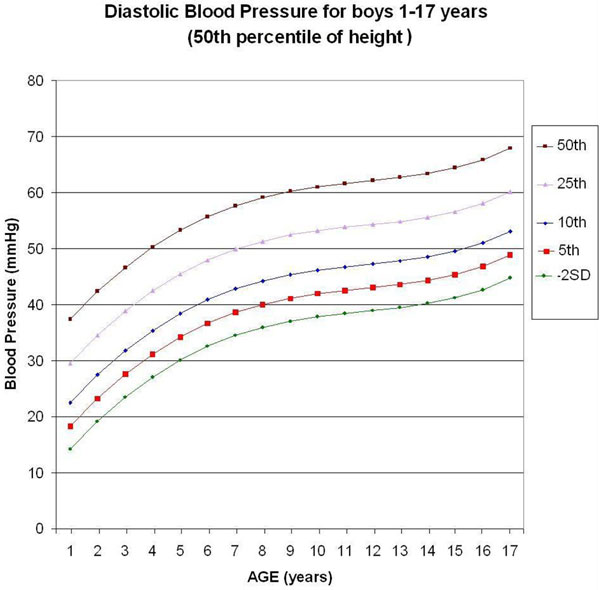
**Graphic update of diastolic blood pressure (DBP) based on the last Report on Blood Pressure in 2004 **[3]**, for boys 1 to 17 years old (50th percentile of height)**. Considering a tolerance of 1 mmHg, the curve of adjustment for height of the DBP for boys does not need any correction in the in the 25.5th to 76th percentile of height.

Five monotonic curves of diastolic BP for boys representing the 50th, 25th, 10th, 5th, and 2.275th (-2SD) percentiles were built (Figure 1). Considering a tolerance of 1 mmHg, the monotonic curve of adjustment for height of the diastolic BP for boys does not need any correction in the 25.5th to 76th percentile of height, but needs maximal correction for the 5th percentile of height (-2.41 mmHg correction).

## Conclusion

The correction of diastolic BP for height is of little significance, and this updated graphic can be used to diagnose low diastolic BP for boys. Clinical studies are necessary to determine the SBP percentile that better represents clinically significant hypotension.
